# Disordered personality traits and psychiatric morbidity in pregnancy: a population-based study

**DOI:** 10.1007/s00737-018-0937-8

**Published:** 2019-01-05

**Authors:** Grace Crowley, Emma Molyneaux, Selina Nath, Kylee Trevillion, Paul Moran, Louise M. Howard

**Affiliations:** 1 Brighton and Sussex Medical School, BSMS Teaching Building, University of Sussex, Falmer, Brighton, BN1 9PX UK; 2 Institute of Psychiatry, Psychology and Neuroscience at King’s College London, Section of Women’s Mental Health, Health Service and Population Research Department, De Crespigny Park, London, SE5 8AF UK; 3 Centre for Academic Mental Health, Department of Population Health Sciences, Bristol Medical School, University of Bristol, Bristol, BS8 2BN UK

**Keywords:** Pregnancy, Perinatal mental health, Personality disorder, Self-harm

## Abstract

This study aims to investigate the characteristics and mental health status of pregnant women with disordered personality traits. A cross-sectional study of a stratified sample of 545 women attending antenatal booking at a South London maternity service was conducted. Disordered personality traits were assessed using the Standardised Assessment of Personality-Abbreviated Scale (SAPAS). Mental disorders were assessed using the Structured Clinical Interview DSM-IV (SCID). Logistic regression was used to model associations, adjusting for confounders. Complete SAPAS data were collected for over 99% of women (*n* = 541). The weighted prevalence of elevated disordered personality traits (SAPAS ≥ 3) was 16.2% (95% CI 12.6–20.5). Women with elevated disordered personality traits were younger, less likely to live alone and more likely to report living in insecure accommodation. Among women with elevated disordered personality traits, the most common mental disorders were anxiety disorders (31.4%) and depressive disorders (17.6%). Each extra item endorsed on the SAPAS was associated with an 82% higher odds of meeting criteria for an Axis I mental disorder (adjusted OR 1.82 (1.42–2.33); *p* < 0.001). Women with elevated disordered personality traits were at significantly increased risk of experiencing thoughts of self-harm (adjusted OR 2.12 (1.33–3.40); *p* = 0.002). Pregnant women with disordered personality traits are a particularly vulnerable population, with multiple psychosocial problems that are likely to require tailored support to ameliorate future health risks for mother and baby.

**Electronic supplementary material:**

The online version of this article (10.1007/s00737-018-0937-8) contains supplementary material, which is available to authorized users.

## Introduction

Maternal mental disorders are important causes of global morbidity and mortality (Atif et al. 2015). The majority of research into maternal mental health has focused on depression and anxiety, and very few studies have examined the association between maternal personality and mental health during the perinatal period (Howard et al. 2014). This is a notable gap in the literature—about 1 in 20 of the general population are affected by personality disorder (PD) (Coid et al. 2006), a condition associated with considerable disease burden (Moran et al. 2016a, b), poor general health (Dixon-Gordon et al. 2018) and raised mortality (Fok et al. 2012).

To our knowledge, only one study has assessed women for PD symptomatology during pregnancy (Börjesson et al. 2005). This Swedish cohort study estimated the prevalence of high PD symptomatology during pregnancy to be 6.4%, using a modified self-reported screening version of the Structured Clinical Interview. High PD symptomatology was associated with elevated levels of other current self-reported psychiatric symptoms during pregnancy and the postnatal period. However, we lack knowledge on the link between disordered personality traits and psychiatric disorders in pregnancy, as well as an understanding about the characteristics of women with disordered personality traits during the perinatal period.

Maternal PD (measured during the postnatal period) has been associated with maladaptive parenting approaches and an increased risk of mental disorders among offspring in later life (Eyden et al. 2016). There is also some evidence from a retrospective case review that, compared to controls, pregnant women with a clinical diagnosis of borderline PD were more likely to experience adverse birth outcomes, such as lower Apgar scores, prematurity and special care nursery referral (Blankley et al. 2015). Examining the prevalence of disordered personality traits in early pregnancy and understanding whether these traits are linked with poorer mental health, alongside other risk factors for poor outcomes, may help inform the development and provision of personalised maternity mental health services.

The primary aim of this study was to examine the characteristics and mental health status of pregnant women identified as having disordered personality traits on a validated screen for PD (Moran et al. 2003). We sought to describe the sociodemographic, obstetric, offspring and health characteristics of these women, then determine whether pregnant women with higher levels of disordered personality traits were more likely to meet diagnostic criteria for an Axis I mental disorder. Secondary aims were to determine whether pregnant women with higher levels of disordered personality traits were more likely to have booked late for their first antenatal appointment (> 12 weeks) or to have current self-harm ideation.

## Materials and methods

### Study population

Cross-sectional data were derived from the “WEll-being in pregNancy stuDY” (WENDY study) (see Howard et al. 2018 for further details on recruitment and sample characteristics). In brief, this is a study examining the prevalence and identification of mental disorders among women receiving antenatal care in a South London maternity service. The sampling frame included all women attending antenatal booking at an inner-city maternity service in South London during the period November 2014–June 2016. Women were potentially eligible if they were aged ≥ 16 years old and answered the Whooley Questions at their first antenatal appointment (a two-item screen for depressive symptoms) (Whooley et al. 1997). Women were excluded from the study if they were < 16 years old, lacked mental capacity to provide informed consent, did not answer the Whooley Questions, had already undergone a comprehensive maternity booking in the UK and/or had a termination or miscarriage between booking and research interview.

### Procedures

Ethical approval was obtained from London Camberwell St. Giles NHS Research Ethics Committee (reference number: 14/LO/0075). Recruitment was performed using a two-phase sampling design (Howard et al. 2018). An advertisement for the study was included in the pre-booking information pack that was sent to all women prior to their antenatal booking appointment. Women were stratified according to whether they responded positively (W+) or negatively (W−) to the two Whooley questions asked by the midwife at their booking appointment. All participants who were W+ and a random sample of W− (using online audit-trailed randomisation) were invited to participate. Researchers interviewed consenting women within 3 weeks of their first antenatal appointment.

### Measures

Researchers administered diagnostic interviews, screening tools and self-reported questionnaires during the research interview (Howard et al. 2018). The measures used for the purposes of this analysis are detailed below.

#### The Standardised Assessment of Personality-Abbreviated Scale

The Standardised Assessment of Personality-Abbreviated Scale (SAPAS) is an interviewer-administered, structured screening tool that identifies individuals at high risk of PD (Moran et al. 2003), consisting of eight items with possible total scores of 0–8. Items cover difficulties in domains such as making and keeping friends, trusting others, impulsivity, worrying and perfectionism. Participants answer the questions in relation to “the way they usually are” when they are their “usual self”. It does not screen for specific sub-types of PD; rather, increasing scores indicate increasing likelihood that an individual has any DSM PD. It was originally validated for use among psychiatric patients, when a cut-point of ≥ 3 provided a sensitivity of 0.94, a specificity of 0.85 and correctly identified DSM-IV PD in 90% of participants with PD (Moran et al. 2003).

#### The Structured Clinical Interview DSM-IV

The Structured Clinical Interview DSM-IV (SCID) is a semi-structured interview based on diagnostic criteria for DSM-IV mental disorders (First et al. 2002; First et al. 1997). The modules of the SCID for Axis I disorders (SCID-I) administered in this study were Mood Episodes, Mood Disorders, Anxiety Disorders and Eating Disorders. The borderline PD sub-section of the SCID for Axis II disorders (SCID-II) was also administered. The SCID was administered by trained researchers, and diagnoses were reached following consensus meetings with co-authors PM and LMH.

#### The Edinburgh Postnatal Depression Scale

The EPDS is a self-reported screen for perinatal depression (Cox et al. 1987). Participants answer the questionnaire in relation to the “last 7 days”. It consists of 10 items, with possible scores of 0–30. Although originally designed to assess postnatal depression, it has been validated for use antenatally (Gibson et al. 2009). For the current analysis, only item 10 on the EPDS was used (*“The thought of harming myself has occurred to me”*) (Cox et al. 1987). This was dichotomised to “Never or hardly ever” and “Sometimes or quite often”. Item 10 dichotomised in this way has been validated as a stand-alone indicator of suicidal ideation in postnatal women (Howard et al. 2011).

The self-reported sociodemographic, obstetric, offspring- and health-related characteristics obtained during the research interview are outlined in Table 1.

**Table 1:** Self-reported sociodemographic, obstetric, offspring- and health-related characteristics obtained during the research interview

Sociodemographic characteristics	Age Ethnicity (Asian, Black, Mixed, White, other) Place of birth (UK, outside UK) Relationship status (married/cohabiting, partner but not cohabiting, separated/divorced/widowed, single) Highest qualification (GCSE/equivalent or less, A-level/equivalent, university degree/relevant professional training) Gross yearly household income Employment status (not working, paid employment, student, other) Living situation (alone, spouse/partner, parent(s)/other family, friends/acquaintance, insecure accommodation/other)
Obstetric and offspring-related characteristics	Whether the current pregnancy had been planned or not Previous termination/s of pregnancy Previous miscarriage/s or stillbirth/s Previous birth/s before 37 weeks gestation Whether an existing child had been assigned to a social worker Whether a referral to social services had been made for the current pregnancy
Health-related characteristics	Current and/or chronic medical condition/s Pre-pregnancy body mass index (BMI) Number of cigarettes smoked daily prior to knowledge of the pregnancy Current and/or chronic mental health condition/s History of self-harm or suicide attempts (assessed from responses during the SCID semi-structured interview) Alcohol consumption (assessed using the Alcohol Use Disorders Identification Test (Babor et al. 2001)) Drug use (assessed using the Drug Use Disorders Identification Test (Berman et al. 2003))

### Statistical analyses

Analyses were performed using Stata 14.0. Data analyses were conducted using survey weighting to account for bias introduced by the over-sampling of Whooley positive and under-sampling of Whooley negative women in the WENDY study (Online Resource 1). SAPAS score was first used as a dichotomous variable to describe the sociodemographic, obstetric, offspring, and health-related characteristic of women with high levels of disordered personality traits (SAPAS scores ≥ 3) compared to those with low SAPAS scores (< 3). Unweighted sample characteristics were also calculated by SAPAS status and are reported in the online resources. Logistic regression was then used to model the associations between SAPAS score and the odds of meeting diagnostic criteria for an Axis I mental disorder, for booking late for the first antenatal appointment and for current self-harm ideation.

Multivariable logistic regression analyses were then run separately for each aim, adjusting for a priori confounders of age and highest qualification. The associations with late antenatal booking and current self-harm ideation were further adjusted for the presence of depression and/or anxiety.

There was very limited missing data for the majority of variables used in these analyses. Missing data is reported in the relevant results tables.

## Results

Five hundred forty-five women participated in the study. The mean gestation of these women at the research interview was 13.5 weeks (range 5–38 weeks, median 12 weeks, interquartile range 11–14 weeks). Of these women, 541 (99.3%) completed the SAPAS and were included in these analyses. Among the 541 women who completed the SAPAS, 130 (24.0%) scored above the conventional cut-point of 3, resulting in a population prevalence estimate of 16.2% (95% confidence interval (CI) 12.6–20.5%) after survey weighting. The median SAPAS score was 2 with an interquartile range of 1–2.

The sample represented a diverse population of women (Online Resource 2). Over half (52.1%) were born outside the UK, and in terms of ethnicity, 52.1% were White, 32.5% Black, 4.6% Asian and 10.8% Mixed/other. Furthermore, a quarter of the women were not currently working, and 14.4% had a gross yearly household income of less than £15,000. Of the women in this sample, 6.3% lived in “insecure/other” accommodation.

Table 2 displays population estimates for sociodemographic characteristics, stratified by SAPAS status. Compared to women with low SAPAS scores (scoring <3), women with high SAPAS scores (scoring ≥ 3) were generally younger. They were also less likely to live alone (4.2%; 95% CI 2.5–6.8% versus 11.7%; 9.3–16.3%) and were more likely to report living in insecure accommodation than women with low SAPAS scores (9.8%; 4.5–20.1% versus 2.9%; 1.4–5.8%), although the confidence intervals for these estimates were wide and overlapping. There were no meaningful differences based on SAPAS screen status for population estimates of any other sociodemographic characteristic.

**Table 2:** Survey-weighted sociodemographic characteristics for all women and by SAPAS status

Characteristics	All women % (95% CI) *N* = 541	SAPAS < 3 % (95% CI) *N* = 411	SAPAS ≥ 3 % (95% CI) *N* = 130	Missing *N* (%)
Age				0 (0)
16–24	5.7 (3.7–8.7)	4.7 (2.8–7.9)	10.8 (5.3–20.8)	
25–29	18.8 (14.8–23.6)	16.3 (12.2–21.5)	31.6 (20.5–45.3)	
30–34	35.2 (30.0–40.7)	35.0 (29.3–41.1)	36.0 (24.2–49.8)	
35–39	32.1 (27.0–37.5)	34.7 (29.1–40.8)	18.4 (10.1–31.3)	
40–49	8.4 (5.6–11.9)	9.2 (6.2–13.5)	3.2 (7.9–12.1)	
Ethnicity				0 (0)
White	55.4 (49.8–60.9)	56.2 (50.0–62.2)	51.4 (38.2–64.4)	
Black	30.6 (25.7–36.0)	29.8 (24.4–35.7)	35.0 (23.6–48.4)	
Asian	4.4 (2.6–7.3)	4.6 (2.6–8.0)	3.4 (0.9–11.9)	
Mixed/other^a^	9.6 (6.8–13.5)	9.5 (6.5–13.8)	10.2 (4.4–22.0)	
Place of birth				0 (0)
UK	49.5 (43.9–55.1)	50.0 (43.9–56.2)	46.6 (33.7–60.0)	
Outside UK	50.5 (44.9–56.1)	50.0 (43.8–56.1)	53.4 (40.0–66.3)	
Relationship status				0 (0)
Married/cohabiting	81.2 (76.6–85.1)	81.5 (76.3–85.8)	79.5 (68.3–87.4)	
Partner but not cohabiting	9.7 (7.0–13.4)	9.9 (6.8–14.1)	9.0 (4.3–17.6)	
Single, separated, divorced or widowed	9.1 (6.4–12.7)	8.6 (5.7–12.7)	11.6 (5.9–21.5)	
Highest qualification^b^				0 (0)
Up to GCSE or equivalent	8.2 (5.7–11.7)	7.8 (5.1–11.7)	10.6 (5.1–20.7)	
A-level or equivalent	27.0 (22.3–32.2)	26.7 (21.6–32.5)	28.6 (18.2–41.9)	
University or relevant professional training	64.8 (59.3–70.0)	65.5 (59.5–71.2)	60.8 (47.5–72.8)	
Gross yearly household income				5 (0.9)
£0–£14,999	11.0 (8.0–14.9)	10.9 (7.6–15.3)	11.6 (5.9–21.5)	
£15,000–£30,999	11.1 (8.0–15.1)	10.6 (7.3–15.0)	13.8 (6.9–25.8)	
£31,000–£45,999	12.7 (9.4–17.0)	11.9 (8.4–16.6)	16.9 (8.7–30.0)	
£46,000–£60,999	13.1 (9.8–17.5)	13.4 (9.7–18.3)	11.6 (5.1–24.3)	
£61,000 or above	32.1 (27.0–37.6)	34.5 (28.8–40.6)	19.9 (11.0–33.2)	
“Rather not say”	20.0 (15.9–24.8)	18.7 (14.4–24.1)	26.2 (16.3–39.3)	
Employment status				0 (0)
Not working	23.3 (18.9–28.3)	22.0 (17.3–27.6)	29.6 (19.1–42.8)	
Paid employment	67.9 (62.5–72.9)	69.2 (63.2–74.6)	60.8 (47.5–72.8)	
Student	4.3 (2.5–7.2)	4.5 (2.5–7.9)	3.2 (7.9–12.1)	
Other^c^	4.6 (2.8–7.5)	4.2 (2.4–7.5)	6.4 (2.4–16.0)	
Living situation				0 (0)
Alone	10.5 (7.6–14.3)	11.7 (8.3–16.3)	4.2 (2.5–6.8)	
Spouse/partner	76.8 (71.8–81.1)	77.2 (71.7–82.0)	74.5 (62.3–83.7)	
Parent(s)/other family	7.0 (4.6–10.4)	6.6 (4.2–10.4)	8.8 (3.7–19.5)	
Friend(s)/acquaintance	1.7 (0.8–3.9)	1.5 (0.6–3.9)	2.8 (0.6–12.6)	
Insecure accommodation/other^d^	4.0 (2.4–6.7)	2.9 (1.4–5.8)	9.8 (4.5–20.1)	

^a^Ethnicity: The most common responses for “Other” were South American, Latin American, Kurdish and Turkish

^b^Highest qualification: The categories were composed of “up to GCSE or equivalent” (which included no formal qualifications or GCSEs/equivalent), “A-level or equivalent” (which included A-levels/equivalent, NVQ level, BTEC level or Higher National Certificate/Diploma) and “University degree or relevant professional training” (which included Bachelors degree, Masters degree, Doctoral degree or relevant professional training)

^c^Employment status: The most common responses for “Other” were self-employed, unable to work due to immigration status, doing freelance work and maternity leave

^d^Insecure accommodation/other: The most common responses in this category were hostel, asylum hostel, homeless, shelter, emergency accommodation, shared house, living with lodgers, living with landlady or a combination of spouse/partner and friends/family

Table 3 displays population estimates of obstetric, offspring- and health-related characteristics, again stratified by SAPAS status. There were no meaningful differences between women with low and high SAPAS scores for any obstetric or offspring-related characteristics. The proportion of women with high SAPAS scores reporting any current and/or chronic mental health condition was over three times that of women with low SAPAS scores (13.2%; 7.2–22.8% versus 3.8%; 2.1–6.5%). Furthermore, the point estimate for the proportion of women with high SAPAS scores who reported a history of self-harm or suicide attempts was over double that of women with low SAPAS scores, although confidence intervals for these estimates were wide and overlapped (15.4%; 8.6–26.0% versus 6.1%; 3.8–9.5%). Estimates for other health-related characteristics did not differ meaningfully between women with low and high SAPAS scores.

**Table 3:** Survey-weighted obstetric, offspring- and health-related characteristics for all women and by SAPAS status

Characteristics	All women % (95% CI) *N* = 541	SAPAS < 3 % (95% CI) *N* = 411	SAPAS ≥ 3 % (95% CI) *N* = 130	Missing *N* (%)
Current pregnancy planned?				0 (0)
Planned	72.9 (67.7–77.5)	73.4 (67.6–78.4)	70.4 (57.6–80.7)	
Unplanned	27.1 (22.5–32.3)	26.6 (21.6–32.4)	29.6 (19.3–42.4)	
Ever had a Termination of Pregnancy?				1 (0.2)
Yes	29.1 (24.3–34.4)	29.7 (24.4–35.6)	26.0 (16.2–39.1)	
No	70.9 (65.6–75.7)	70.3 (64.4–75.6)	74.0 (60.9–83.8)	
Ever had a Miscarriage or Stillbirth?				2 (0.4)
Yes	32.6 (27.6–38.1)	32.7 (27.2–38.8)	31.9 (20.7–45.5)	
No	67.4 (61.9–72.4)	67.3 (61.2–72.8)	68.1 (54.5–79.3)	
Any of their children born at <37 Weeks Gestation?				0 (0)
Yes	11.7 (7.4–17.8)	12.4 (7.7–19.4)	7.4 (2.1–23.2)	
No	88.4 (82.2–92.6)	87.6 (80.6–92.3)	92.6 (76.7–97.9)	
Any of their children have a social worker or social services referral for this pregnancy?				2 (0.4)
Yes	2.0 (1.0–4.0)	1.7 (0.7–4.0)	3.3 (0.8–12.3)	
No	98.0 (96.0–99.0)	98.3 (96.0–99.3)	96.7 (87.7–99.1)	
Any current and/or chronic medical condition/s				1 (0.2)
Yes	41.7 (36.3–47.3)	41.1 (35.2–47.3)	45.0 (32.3–58.4)	
No	58.3 (52.7–63.7)	58.9 (52.7–64.8)	55.0 (41.6–67.7)	
Pre-pregnancy BMI				123 (22.6)
Underweight (≤ 18.4 kg/m)	6.0 (3.6–9.7)	5.8 (3.3–9.9)	7.2 (3.4–20.0)	
Healthy (18.5–24.9 kg/m)	65.7 (59.5–71.4)	64.3 (57.4–70.6)	73.3 (58.3–84.4)	
Overweight (25.0–29.9 kg/m)	18.3 (13.9–23.6)	20.5 (15.5–26.6)	6.2 (2.3–15.4)	
Obese (≥ 30.0 kg/m)	10.1 (6.8–14.5)	9.4 (6.1–14.3)	13.4 (5.8–27.8)	
Daily number of cigarettes smoked prior to knowledge of the pregnancy				0 (0)
None	90.7 (87.0–93.4)	91.3 (87.2–94.1)	87.8 (76.7–94.1)	
1–5	5.8 (3.7–9.0)	5.6 (3.4–9.2)	7.0 (2.8–16.3)	
6+	3.5 (1.9–6.1)	3.1 (1.6–6.0)	5.2 (1.6–15.8)	
Harmful or hazardous alcohol consumption in last year				13 (2.4)
Yes	4.5 (2.7–7.5)	3.8 (2.1–7.1)	8.0 (3.0–19.5)	
No	95.5 (92.5–97.3)	96.2 (92.9–97.9)	92.0 (80.5–97.0)	
Drug-related problem in last year				12 (2.2)
Yes	6.9 (4.5–10.2)	6.8 (4.3–10.7)	7.0 (2.7–16.6)	
No	93.1(89.8–95.5)	93.2 (89.3–95.7)	93.0 (83.4–97.3)	
Any current and/or chronic mental health condition/s				1 (0.2)
Yes	5.3 (3.5–7.9)	3.8 (2.1–6.5)	13.2 (7.2–22.8)	
No	94.7 (92.1–96.5)	96.3 (93.5–97.9)	86.8 (77.2–92.8)	
History of self-harm or suicide attempts				1 (0.2)
Yes	7.6 (5.3–10.8)	6.1 (3.8–9.6)	15.4 (8.6–26.0)	
No	92.4 (89.2–94.7)	93.9 (90.4–96.2)	84.6 (74.0–91.4)	

Table 4 displays the results of regression analyses for the association between SAPAS score and Axis I mental disorder. Following survey weighting, the population prevalence estimate for meeting diagnostic criteria for an Axis I mental disorder was 23.6% (95% CI 19.3–28.5). A one-point increase in SAPAS score was associated with an 82% increase in the odds of meeting criteria for an Axis I mental disorder (age- and education-adjusted OR 1.82, 95% CI 1.42–2.33, *p* < 0.001).

**Table 4:** Logistic regression analyses for the association between SAPAS score and meeting diagnostic criteria for any Axis I mental disorder, using the SAPAS as a continuous variable

Meets diagnostic criteria for any Axis I mental disorder^a^		Unadjusted model (*n* = 517)		Adjusted model (age and highest qualification) (*n* = 517)	
		OR (95% CI)	*p*	OR (95% CI)	*p*
SAPAS score		1.84 (1.46–2.31)	< 0.001	1.82 (1.42–2.33)	< 0.001
Age (years)	16–24			0.15 (0.05–0.49)	0.002
	25–29			0.15 (0.05–0.44)	0.001
	30–34			0.19 (0.06–0.57)	0.003
	35–39			0.04 (0.01–0.13)	< 0.001
	40–49			Reference group	–
Highest qualification	Up to GCSE or equivalent			1.13 (0.41–3.13)	0.81
	A-level or equivalent			1.05 (0.41–2.74)	0.91
	University degree or relevant professional training			Reference group	–

Analyses conducted using survey-weighted data

^a^Diagnostic criteria for any Axis I mental disorder includes the following: major depressive disorder, current major depressive episode, mixed anxiety and depressive disorder, GAD, OCD, panic disorder, agoraphobia without history of panic disorder, social phobia, specific phobia, PTSD, anorexia nervosa, atypical anorexia nervosa, bulimia nervosa, binge eating disorder, purging disorder, other specified feeding and eating disorder, bipolar I disorder, bipolar II disorder, current manic episode and current hypomanic episode

The overall response rate for all SCID modules was 94.9% (*n* = 517). Twenty-one (3.9%) women declined to answer the PTSD module of SCID-I, and the prevalence of PTSD reported in the sample was low (0.8%; 95% CI 0.3–2.1%). Survey-weighted prevalence estimates were calculated for Axis I disorders and borderline PD, stratified by SAPAS status. The most prevalent disorders were anxiety (14.7%; 95% CI 11.2–19.0) and depressive disorders (10.2%; 7.6–13.5%). The prevalence of depressive disorders among women with high SAPAS scores was double that among women with low SAPAS scores (17.6%; 10.9–27.1% versus 8.8%; 6.1–12.4%), but confidence intervals for these estimates were wide and overlapped. The prevalence of anxiety disorders among women with high SAPAS scores was nearly triple that among women with low SAPAS scores (31.4%; 20.6–44.9% versus 11.5%; 8.2–15.9%). There was no evidence that the prevalence of OCD, any eating disorder or any “other” Axis I disorder differed among women with high or low SAPAS scores; however, the prevalence of these disorders in the sample was low. The prevalence of borderline PD (as measured by the SCID-II borderline PD sub-section) was 0.7% (95% CI 0.2–2.0%), and as anticipated, was substantially higher among women with high SAPAS scores (4.2%; 1.4–11.9%) than women with low SAPAS scores (0.1%; 0.0–0.3%).

The association between SAPAS score and booking late for antenatal care was then examined. The population estimate for booking late for the first antenatal appointment was 15.0% (95% CI 11.5–19.4%), 18.1% (10.2–30.3%) among women with high SAPAS scores (≥ 3) and 14.4% (10.6–19.3%) among women with low SAPAS scores (<3). There was no evidence of an association between SAPAS score and late booking in the unadjusted model (OR 1.16, 95% CI 0.90–1.49, *p* = 0.26) or following adjustment for age and education (OR 1.08, 95% CI 0.83–1.40, *p* = 0.56). Additional adjustment for presence of an anxiety and/or depressive disorder had no meaningful impact on the adjusted estimate.

The weighted population prevalence estimate of current self-harm ideation was 2.3% (95% CI 1.2–4.3%). Among women with high SAPAS scores, the population prevalence estimate was 9.0% (95% CI 4.4–17.7%), compared with 1.0% (95% CI 0.3–3.2%) among women with low SAPAS scores. Table 5 displays the results of regression analyses for the association between SAPAS score and self-harm ideation. For every one-point increase in SAPAS score, there was over double the odds of reporting current self-harm ideation, independent of age and highest qualifications (OR 2.12, 95% CI 1.33–3.40, *p* = 0.002). Adjustment for anxiety and depression slightly attenuated the size of association, but it remained statistically significant (OR 1.90, 95% CI 1.03–3.52, *p* = 0.04). To our knowledge, this is the first time such an association has been detected in a perinatal sample and this finding highlights the importance of identifying disordered personality traits as part of clinical risk assessment.

**Table 5:** Logistic regression analyses for the association between SAPAS score and reporting self-harm ideation, using the SAPAS as a continuous variable

		Unadjusted model (*n* = 535)		Adjusted model 1 Adjusted for age and highest qualification (*n* = 535)		Adjusted model 2 Additionally adjusted for anxiety and depression^a^ (*n* = 532)	
		OR (95% CI)	*p*	OR (95% CI)	*p*	OR (95% CI)	*p*
SAPAS score		2.22 (1.38–3.58)	0.001	2.12 (1.33–3.40)	0.002	1.90 (1.03–3.52)	0.04
Age (years)	16–24			0.82 (0.15–4.45)	0.82	1.24 (0.25–6.13)	0.79
	25–29			1.31 (0.20–8.36)	0.78	1.95 (0.25–14.96)	0.52
	30–34			0.22 (0.03–1.45)	0.11	0.35 (0.05–2.56)	0.30
	35–39			0.20 (0.01–3.26)	0.26	0.41 (0.25–6.55)	0.52
	40–49			Reference group	–	Reference group	–
Highest qualification	Up to GCSE or equivalent			2.28 (0.48–10.78)	0.30	2.54 (0.51–12.71)	0.26
	A-level or equivalent			1.70 (0.42–6.89)	0.46	1.77 (0.45–7.02)	0.41
	University or equivalent			Reference group	–	Reference group	–
Depressive or anxiety disorder^a^	Yes					3.47 (0.52-23.06)	0.20
	No					Reference group	–

Analyses conducted using survey-weighted data. PTSD and OCD were not included because, although they were classified as anxiety disorders in the DSM-IV, they are now recognised as separate categories in the DSM-V (American Psychiatric Association 2013)

^a^Diagnostic criteria for depressive or anxiety disorder includes the following: major depressive disorder, current major depressive episode, mixed anxiety and depressive disorder, GAD, panic disorder, agoraphobia without history of panic disorder, social phobia, and specific phobia

## Discussion

In this representative sample of pregnant women from South London, the presence of disordered personality traits was independently associated with common mental disorders and thoughts of self-harm. These associations remained after adjustment for age and education. By applying a recommended cut-point on the SAPAS screening scale, we determined that just under one in six women reported disordered personality traits in early pregnancy. These women were younger and may be more likely to live in insecure accommodation and report previous self-harm and suicide attempts.

The finding that women with elevated disordered personality traits were younger is in keeping with recent English survey data showing an apparent age-related decline in the prevalence of PD (Moran et al. 2016b). Women with high SAPAS scores were also less likely to live alone but more likely to live in an insecure form of accommodation such as asylum hostels and homeless shelters. Although the confidence intervals overlapped for these estimates, the lack of significance is likely to be due to underpowered subgroup analyses. These findings are consistent with other literature showing that individuals with PD are over-represented in homeless populations and more likely to experience financial and social disadvantage over the life course (Moran et al. 2016a; Skodol 2018).

Multivariable analyses also provided evidence for the existence of an association between increasing SAPAS scores and the presence of Axis I mental disorders during pregnancy, independent of age and qualifications. This is consistent with previous studies that have found high rates of mental disorder comorbidity among individuals with PD in the general population (Tomko et al. 2014). Multivariable analyses also identified an association with reporting self-harm ideation, which remained after adjusting for current depressive and anxiety disorders. Prevalence estimates suggest that just under one in ten pregnant women with high levels of disordered personality traits have current thoughts of self-harm, compared with one in a hundred pregnant women with low levels of disordered personality traits.

### Strengths and limitations

There was very little missing data for the majority of variables, including almost 100% complete data for the assessment of disordered personality traits. However, larger amounts of missing data for some variables (particularly PTSD) may have led to an underestimation of the overall prevalence of Axis I mental disorders in the sample.

Over half the women in the WENDY sample were born outside the UK, and there was a wide range of reported ethnicities and incomes, reflecting the diversity characterising the maternity population in South London. The current evidence for perinatal mental disorders among Black minority ethnic women in the UK is extremely limited (National Mental Health Development Unit 2011). Therefore, results from the WENDY study are likely to provide new insights into the characteristics of pregnant women who are otherwise under-researched. Another strength of the study was that both screening and diagnostic information was collected. Previous studies of perinatal personality dysfunction and comorbid mental health problems have relied on self-reported questionnaires and are therefore prone to information bias, as opposed to measuring mental disorders using a structured interview (Börjesson et al. 2005; Hudson et al. 2017).

The findings need to be considered in light of several limitations. Our participation rate (33%) was suboptimal, but the recruited sample was representative of the local population and was sufficiently large enough to fulfil the aims of this study. Due to the cross-sectional nature of the study design, we cannot rule out reverse causality as an explanation for some of the associations. In addition, the assessment of personality could have been biased by present mental state. Some of the personality traits measured by the SAPAS overlap with symptoms of Axis I mental disorders, for instance, “*Are you normally a worrier?*” represents a diagnostic feature of generalised anxiety disorder (American Psychiatric Association 2013). Therefore, it may be difficult to assess personality pathology if there are concurrent Axis I mental disorders, which is a limitation of this study. Furthermore, our use of a brief screen for PD limited the depth of data that might have been obtained had we used a diagnostic interview for all PDs. However, the brevity of the screen minimised respondent burden and allowed us to collect data on nearly the entire sample. It is important to highlight that the majority of women scoring above the cutoff (≥ 3) on the SAPAS would not meet diagnostic criteria for PD and that using a slightly higher cutoff on the SAPAS (≥ 4) as an additional analysis, we obtained a much lower population prevalence estimate for disordered personality traits (4.4% versus 16%). The classification of PD is the subject of much debate and personality is considered to be best captured as a continuous dimension in the general population (Kim and Tyrer 2010). While important issues with the construct of PD remain, by treating the SAPAS as a continuous variable in the regression models, we were able to explore the risk of mental health problems associated with a subthreshold increase in the number of personality traits as recorded on the SAPAS.

### Implications for clinical practice

The results of our study suggest that healthcare professionals should be aware that pregnant women with elevated disordered personality traits are likely to need increased support for common mental disorders and support for other vulnerabilities, potentially including thoughts of self-harm and other factors related to their social situation. We found no evidence that disordered personality traits were associated with booking late for antenatal care, which highlights the potential to provide timely support for these women during pregnancy. It is increasingly clear that personality features are of prognostic significance in the treatment of anxiety and depression (Goddard et al. 2015) and for mother-infant interactions (Nath et al., submitted), and interventions therefore need to be appropriately tailored for women experiencing these traits.

## Conclusions

Women with disordered personality traits in early pregnancy are vulnerable to poor mental health, thoughts of self-harm and possible additional difficulties relating to insecure accommodation. Future research should explore their pregnancy and parenting needs to inform appropriate service provision.

## Electronic supplementary material

ESM 1 (DOCX 47 kb)

## References

[CR1] American Psychiatric Association (2013). Diagnostic and Statistical Manual Of Mental Disorders.

[CR2] Atif N, Lovell K, Rahman A (2015). Maternal mental health: the missing “m” in the global maternal and child health agenda. Semin Perinatol.

[CR3] Babor F, Higgins-Biddle J, Saunders J, Monteiro M (2001) The Alcohol Use Disorders Identification Test guidelines for use in primary care second edition [Internet]. World Health Organization Department of Mental Health and Substance Dependence. [cited 2017 Jul 7]. Available from: https://www.alcohollearningcentre.org.uk/_assets/WHO_-_AUDIT.pdf

[CR4] Berman A, Bergman H, Palmstierna T, Frans Schlyter F (2003) The Drug Use Disorders Identification Test manual [Internet]. Karolinska Institutet, Department of Clinical Neuroscience. [cited 2017 Jul 7]. Available from: https://www.paihdelinkki.fi/sites/default/files/duditmanual.pdf

[CR5] Blankley G, Galbally M, Snellen M, Power J, Lewis AJ (2015). Borderline personality disorder in the perinatal period: early infant and maternal outcomes. Australas Psychiatry.

[CR6] Börjesson K, Ruppert S, Bågedahl-Strindlund M (2005). A longitudinal study of psychiatric symptoms in primiparous women: relation to personality disorders and sociodemographic factors. Arch Womens Ment Health.

[CR7] Coid J, Yang M, Tyrer P, Roberts A, Ullrich S (2006). Prevalence and correlates of personality disorder in Great Britain. Br J Psychiatry.

[CR8] Cox JL, Holden JM, Sagovsky R (1987). Detection of postnatal depression. Development of the 10-item Edinburgh Postnatal Depression Scale. Br J Psychiatry.

[CR9] Dixon-Gordon KL, Conkey LC, Whalen DJ (2018). Recent advances in understanding physical health problems in personality disorders. Curr Opin Psychol.

[CR10] Eyden J, Winsper C, Wolke D, Broome MR, MacCallum F (2016). A systematic review of the parenting and outcomes experienced by offspring of mothers with borderline personality pathology: potential mechanisms and clinical implications. Clin Psychol Rev.

[CR11] Fok ML-Y, Hayes RD, Chang C-K, Stewart R, Callard FJ, Moran P (2012). Life expectancy at birth and all-cause mortality among people with personality disorder. J Psychosom Res.

[CR12] First MB, Gibbon M, Spitzer RL, Williams JBW, Benjamin LS (1997) Clinical interview for DSM-IV Axis II Personality Disorders (SCID-II). Washington D.C: American Psychiatric Press, Inc.

[CR13] First MB, Spitzer RL, Gibbon M, Williams JBW (2002). Structured Clinical Interview for DSM-IV-TR Axis I Disorders, Research Version (SCID-I/NP).

[CR14] Gibson J, McKenzie-McHarg K, Shakespeare J, Price J, Gray R (2009). A systematic review of studies validating the Edinburgh Postnatal Depression Scale in antepartum and postpartum women. Acta Psychiatr Scand.

[CR15] Goddard E, Wingrove J, Moran P (2015). The impact of comorbid personality difficulties on response to IAPT treatment for depression and anxiety. Behav Res Ther.

[CR16] Howard LM, Flach C, Mehay A, Sharp D, Tylee A (2011). The prevalence of suicidal ideation identified by the Edinburgh Postnatal Depression Scale in postpartum women in primary care: findings from the RESPOND trial. BMC Pregnancy Childbirth.

[CR17] Howard LM, Molyneaux E, Dennis C-L, Rochat T, Stein A, Milgrom J (2014). Non-psychotic mental disorders in the perinatal period. Lancet.

[CR18] Howard LM, Ryan EG, Trevillion K, Anderson F, Bick D, Bye A, Byford S, O’Connor S, Sands P, Demilew J, Milgrom J, Pickles A (2018). Accuracy of the Whooley questions and the Edinburgh Postnatal Depression Scale in identifying depression and other mental disorders in early pregnancy. Br J Psychiatry.

[CR19] Hudson C, Spry E, Borschmann R, Becker D, Moran P, Olsson C, Coffey C, Romaniuk H, Bayer JK, Patton GC (2017). Preconception personality disorder and antenatal maternal mental health: a population-based cohort study. J Affect Disord.

[CR20] Kim Y-R, Tyrer P (2010). Controversies surrounding classification of personality disorder. Psychiatry Investig.

[CR21] Moran P, Leese M, Lee T, Walters P, Thornicroft G, Mann A (2003). Standardised Assessment of Personality-Abbreviated Scale (SAPAS): preliminary validation of a brief screen for personality disorder. Br J Psychiatry.

[CR22] Moran P, Romaniuk H, Coffey C, Chanen A, Degenhardt L, Borschmann R, Patton GC (2016). The influence of personality disorder on the future mental health and social adjustment of young adults: a population-based, longitudinal cohort study. Lancet Psychiatry.

[CR23] Moran P, Rooney K, Tyrer P, Coid J. (2016b). “Chapter 7: Personality disorder” in McManus S, Bebbington P, Jenkins R, Brugha T. (eds.) Mental health and wellbeing in England: Adult Psychiatric Morbidity Survey 2014. Leeds: NHS Digital

[CR24] National Mental Health Development Unit. National Perinatal Mental Health Project. Perinatal mental health of black and minority ethnic women: a review of current provision in England, Scotland and Wales. [Internet]. 2011 [cited 2017 Aug 23]. Available from: https://www.gov.uk/government/uploads/system/uploads/attachment_data/file/215718/dh_124880.pdf

[CR25] Skodol AE (2018). Impact of personality pathology on psychosocial functioning. Curr Opin Psychol.

[CR26] Tomko RL, Trull TJ, Wood PK, Sher KJ (2014). Characteristics of borderline personality disorder in a community sample: comorbidity, treatment utilization, and general functioning. J Pers Disord.

[CR27] Whooley MA, Avins AL, Miranda J, Browner WS (1997). Case-finding instruments for depression. Two questions are as good as many. J Gen Intern Med.

