# Supplementary material for: Disordered personality traits and psychiatric morbidity in pregnancy: a population-based study
Source: Arch Womens Ment Health. 2019 Jan 5;23(1):43–52. doi: 10.1007/s00737-018-0937-8 (PMC6987086; doi:10.1007/s00737-018-0937-8)
Supplement: Supplementary file 1 — (DOCX 47 kb) [file 737_2018_937_MOESM1_ESM.docx]

**Online Resource 1: Sampling weights**

Weights were created based on the number of Whooley positive and Whooley negative women included in the study (287 and 258, respectively) and the number of Whooley positive and Whooley negative women in the sampling frame (906 and 9057, respectively) (Howard et al. 2018). The weights applied to the analyses were therefore 906/287 for Whooley positive women and 9057/258 for Whooley negative women (Howard et al. 2018).

**Online Resource 2: Unweighted sociodemographic characteristics for all women and by SAPAS status**

| Characteristics | All women  N (%)  n=541 | SAPAS <3  N (%)  n=411 | SAPAS ≥3  N (%)  n=130 | P^5^ | Missing  n(%) |
| --- | --- | --- | --- | --- | --- |
| Age | | | | <0.001 | 0(0) |
| 16-24 | 57 (10.5) | 33 (8.0) | 24 (18.5) |  |  |
| 25-29 | 101 (18.7) | 63 (15.3) | 38 (29.2) |  |  |
| 30-34 | 177 (32.7) | 137 (33.3) | 40 (30.8) |  |  |
| 35-39 | 161 (29.8) | 139 (33.8) | 22 (16.9) |  |  |
| 40-49 | 45 (8.3) | 39 (9.5) | 6 (4.6) |  |  |
| Ethnicity | | | | 0.012 | 0(0) |
| White | 282 (52.1) | 225 (54.7) | 57 (43.9) |  |  |
| Black | 176 (32.5) | 121 (29.4) | 55 (42.3) |  |  |
| Asian | 25 (4.6) | 18 (4.4) | 7 (5.4) |  |  |
| Mixed/other^1^ | 58 (10.8) | 47 (11.5) | 11 (8.5) |  |  |
| Place of birth | | | | 0.063 | 0(0) |
| UK | 259 (47.9) | 206 (50.1) | 53 (40.8) |  |  |
| Outside UK | 282 (52.1) | 205 (49.9) | 77 (59.2) |  |  |
| Relationship Status | | | | <0.001 | 0(0) |
| Married/cohabiting | 389 (71.9) | 312 (75.9) | 77 (59.2) |  |  |
| Partner but not cohabiting | 81 (15.0) | 56 (13.6) | 25 (19.2) |  |  |
| Single, separated, divorced or widowed | 71 (13.2) | 43 (10.4) | 28 (21.5) |  |  |
| Highest Qualification^2^ | | | | 0.012 | 0(0) |
| Up to GCSE or equivalent | 65 (12.0) | 42 (10.2) | 23 (17.7) |  |  |
| A-level or equivalent | 154 (28.5) | 111 (27.0) | 43 (33.1) |  |  |
| University or relevant professional training | 322 (59.5) | 258 (62.8) | 64 (49.2) |  |  |
| Gross Yearly Household Income | | | | <0.001 | 5 (0.9) |
| £0-£14999 | 77 (14.4) | 49 (12.0) | 28 (21.7) |  |  |
| £15000-£30999 | 70 (13.1) | 51 (12.5) | 19 (14.7) |  |  |
| £31000-£45999 | 60 (11.2) | 46 (11.3) | 14 (10.9) |  |  |
| £46000-£60999 | 63 (11.8) | 55 (13.5) | 8 (6.2) |  |  |
| £61000 or above | 144 (26.9) | 125 (30.7) | 19 (14.7) |  |  |
| “Rather not say” | 122 (22.8) | 81 (19.9) | 41 (31.8) |  |  |
| Employment Status | | | | <0.001 | 0(0) |
| Not working | 139 (25.7) | 91 (22.1) | 48 (36.9) |  |  |
| Paid employment | 348 (64.3) | 284 (69.1) | 64 (49.2) |  |  |
| Student | 22 (4.1) | 16 (3.9) | 6 (4.6) |  |  |
| Other^3^ | 32 (5.9) | 20 (4.9) | 12 (9.2) |  |  |
| Living Situation | | | | <0.001 | 0(0) |
| Alone | 74 (13.7) | 53 (12.9) | 21 (16.2) |  |  |
| Spouse/partner | 373 (69.0) | 301 (73.2) | 72 (55.4) |  |  |
| Parent(s)/other family | 46 (8.5) | 32 (7.8) | 14 (10.8) |  |  |
| Friend(s)/acquaintance | 14 (2.6) | 10 (2.4) | 4 (3.1) |  |  |
| Insecure accommodation/ other^4^ | 34 (6.3) | 15 (3.7) | 19 (14.6) |  |  |

**^1^Ethnicity:** The most common responses for “Other” were South American, Latin American, Kurdish and Turkish.

**^2^Highest qualification:** The categories were composed of “up to GCSE or equivalent” (which included no formal qualifications or GCSEs/equivalent), “A-level or equivalent” (which included A-levels/equivalent, NVQ level, BTEC level or Higher National Certificate/Diploma) and “University degree or relevant professional training” (which included Bachelors degree, Masters degree, Doctoral degree or relevant professional training).

**^3^Employment status:** The most common responses for “Other” were self-employed, unable to work due to immigration status, doing freelance work and maternity leave.

**^4^Insecure living situation/other:** The most common responses in this category were hostel, asylum hostel, homeless, shelter, emergency accommodation, shared house, living with lodgers, living with landlady or a combination of spouse/partner and friends/family.

^5^P value based on Chi-square test

**Online Resource 3: Unweighted obstetric-, offspring- and health- related characteristics for all women and by SAPAS status**

| Characteristics | All women  N (%)  n=541 | SAPAS <3  N (%)  n=411 | SAPAS ≥3  N (%)  n=130 | P^1^ | Missing n(%) |
| --- | --- | --- | --- | --- | --- |
| Current pregnancy planned? | | | | 0.007 | 0(0) |
| Planned | 353 (65.3) | 281 (68.4) | 72 (55.4) |  |  |
| Unplanned | 188 (34.8) | 130 (31.6) | 48 (44.6) |  |  |
| Ever had a Termination of Pregnancy? | | | | 0.935 | 1(0.2) |
| Yes | 169 (31.3) | 129 (31.4) | 40 (31.0) |  |  |
| No | 371 (68.7) | 282 (68.6) | 89 (69.0) |  |  |
| Ever had a Miscarriage or Stillbirth? | | | | 0.833 | 2(0.4) |
| Yes | 167 (31.0) | 128 (31.2) | 39 (30.2) |  |  |
| No | 372 (69.0) | 282 (68.8) | 90 (69.8) |  |  |
| Any of their children Born at <37 Weeks Gestation? | | | | 0.382 | 0(0) |
| Yes | 27 (9.8) | 19 (9.0) | 8 (12.7) |  |  |
| No | 248 (90.2) | 193 (91.0) | 55 (87.3) |  |  |
| Any of their children have a social worker or social services referral for this pregnancy? | | | | 0.596 | 2(0.4) |
| Yes | 21 (3.9) | 15 (3.7) | 6 (4.7) |  |  |
| No | 518 (96.1) | 396 (96.4) | 122 (95.3) |  |  |
| Any current and/or chronic medical condition/s | | | | 0.138 | 0(0) |
| Yes | 240 (44.4) | 175 (42.6) | 65 (50.0) |  |  |
| No | 301 (55.6) | 236 (57.4) | 65 (50.0) |  |  |
| Pre-pregnancy BMI | | | | 0.298 | 122(22.6) |
| Underweight (≤18.4 kg/m) | 29 (6.9) | 21 (6.6) | 8 (8.1) |  |  |
| Healthy (18.5-24.9 kg/m) | 265 (63.3) | 200 (62.5) | 65 (65.7) |  |  |
| Overweight (25.0-29.9 kg/m) | 85 (20.3) | 71 (22.2) | 14 (14.1) |  |  |
| Obese (≥30.0 kg/m) | 40 (9.6) | 28 (8.8) | 12 (12.1) |  |  |
| Daily number of cigarettes smoked prior to knowledge of the pregnancy | | | | 0.116 | 0(0) |
| None | 474 (87.6) | 365 (88.8) | 109 (83.9) |  |  |
| 1-5 | 40 (7.4) | 25 (6.1) | 15 (11.5) |  |  |
| 6+ | 27 (5.0) | 21 (5.1) | 6 (4.6) |  |  |
| Harmful or hazardous alcohol consumption in last year | | | | 0.247 | 13(2.4) |
| Yes | 27 (5.1) | 18 (4.5) | 9 (7.1) |  |  |
| No | 501 (94.9) | 383 (95.5) | 118 (92.9) |  |  |
| Drug-related problem in last year | | | | 0.314 | 12(2.2) |
| Yes | 47 (8.9) | 33 (8.2) | 14 (11.1) |  |  |
| No | 482 (91.1) | 370 (91.8) | 112 (88.9) |  |  |
| Any current and/or chronic mental health condition/s | | | | <0.001 | 1(0.2) |
| Yes | 73 (13.5) | 37 (9.0) | 36 (27.7) |  |  |
| No | 467 (86.5) | 373 (91.0) | 94 (72.3) |  |  |
| History of self-harm or suicide attempts | | | | <0.001 | 1(0.2) |
| Yes | 75 (13.9) | 38 (9.3) | 37 (28.5) |  |  |
| No | 465 (86.1) | 372 (90.7) | 93 (71.5) |  |  |

^1^P value based on Chi-square test

**Online Resource 4: Survey-weighted prevalence of Axis I mental disorders and Borderline PD for all women and by SAPAS status**

| **SCID diagnosis** | **All women % (95%CI)** | **SAPAS <3 % (95%CI)** | **SAPAS ≥3 % (95%CI)** | **Missing,**  **n (%)** |
| --- | --- | --- | --- | --- |
| Axis I mental disorder^1^ | 23.6 (19.3-28.5) | 19.0 (14.8-24.2) | 47.4 (34.2-60.9) | 28 (5.1) |
| Depressive disorder^2^ | 10.2 (7.6-13.5) | 8.8 (6.1-12.4) | 17.6 (10.9-27.1) | 6 (1.1) |
| Anxiety disorder^3^ | 14.7 (11.2-19.0) | 11.5 (8.2-15.9) | 31.4 (20.6-44.9) | 6 (1.1) |
| PTSD | 0.8 (0.3-2.1) | 0.1 (0.0-0.3) | 4.6 (1.6-12.3) | 21 (3.9) |
| OCD | 2.2 (1.1-4.5) | 2.0 (0.8-4.6) | 3.6 (1.0-11.8) | 0 (0) |
| Eating disorder^4^ | 1.5 (0.6-3.4) | 1.6 (0.6-3.9) | 1.0 (0.4-2.5) | 2 (0.4) |
| ‘Other’ Axis I disorder^5^ | 0.0 (0.0-0.3) | 0.0 (0.0-0.3) | 0.2 (0.0-1.5) | 5 (0.9) |
| Borderline PD | 0.7 (0.2-2.0) | 0.1 (0.0-0.3) | 4.2 (1.4-11.9) | 1 (0.2) |

**Analyses conducted using survey weighted data**

**^1^Diagnostic criteria for Axis I mental disorder includes:** major depressive disorder, current major depressive episode, current mixed anxiety and depressive disorder, GAD, OCD, panic disorder, agoraphobia without history of panic disorder, social phobia, specific phobia, PTSD, anorexia nervosa, atypical anorexia nervosa, bulimia nervosa, binge eating disorder, purging disorder, other specified feeding and eating disorder, bipolar I disorder, bipolar II disorder, current manic episode, current hypomanic episode.

**^2^Diagnostic criteria for any depressive disorder includes:** major depressive disorder, current major depressive episode, mixed anxiety and depressive disorder.

**^3^Diagnostic criteria for any anxiety disorder includes:** GAD, panic disorder, agoraphobia without history of panic disorder, social phobia, and specific phobia.

PTSD and OCD were not included under “any anxiety disorder” because, although they were classified as anxiety disorders in the DSM-IV, they are now recognised as separate categories in the DSM-V (American Psychiatric Association 2013).

**^4^Diagnostic criteria for any eating disorder includes:** anorexia nervosa, atypical anorexia nervosa, bulimia nervosa, binge eating disorder, purging disorder and other specified feeding and eating disorder.

**^5^Diagnostic criteria for any ‘other’ Axis I mental disorder includes:** bipolar I disorder, bipolar II disorder, current manic episode and current hypomanic episode.
